# Relationship between the Usage of Long-Lasting Insecticide-Treated Bed Nets (LLITNs) and Malaria Prevalence among School-Age Children in Southwestern Nigeria

**DOI:** 10.1155/2021/8821397

**Published:** 2021-03-25

**Authors:** Adejumoke Oluwatosin Omonijo, Adetunji Omonijo, Hillary Iwegbunem Okoh, Azeez Oyemomi Ibrahim

**Affiliations:** ^1^Department of Animal and Environmental Biology, Federal University Oye Ekiti State, Oye Ekiti, Nigeria; ^2^Department of Family Medicine, Federal Teaching Hospital, Ido Ekiti, Ekiti State, Nigeria

## Abstract

**Purpose:**

The usage of LLITNs in malaria vector control has resulted in the reduction in malaria deaths among higher-risk groups (pregnant women and under-fives). However, there exists asymptomatic infection among older children, thereby making them a reservoir of malaria transmission. This study aimed at assessing the impact of LLITN usage on malaria prevalence among school-age children (SAC) in Ekiti, South Western Nigeria.

**Methods:**

Cross-sectional, two-stage cluster sampling technique was used to collect data from SAC during May and June 2017. A total of 1313 (Oye LGA: 657 and Ikole LGA: 656) SAC in selected public primary schools participated in the study. Sociodemographic information as well as data on LLITN usage the previous night was obtained using pretested, semistructured questionnaires adapted from the standardized Malaria Indicator Survey (MIS) tools. Malaria infection was diagnosed by using the rapid diagnostic test (RDT) on blood samples that were collected by finger prick from each child. Data were analyzed using the Statistical Package for Social Sciences (SPSS) for Windows software version 26 (SPSS Inc., Chicago, IL, USA).

**Results:**

Usage of LLITNs among SAC was significantly higher in Ikole LGA than in Oye (*p* < 0.001). Socioeconomic factors (access to electricity, mother's occupation, and household size) showed significant associations with LLITN usage (*p* < 0.001) in both Oye and Ikole LGAs. Malaria prevalence was significantly low among SAC utilizing LLITNs in both Oye and Ikole LGAs (*p* < 0.001). There was a significant association between gender and malaria prevalence among SAC with males having higher prevalence than females (*p* < 0.001). Socioeconomic factors were significantly associated with malaria prevalence in both LGAs (*p* < 0.001).

**Conclusion:**

The usage of LLITNs caused a significant reduction in malaria prevalence among the school-age children in the study areas; hence, sensitization on usage should be scaled up towards malaria elimination.

## 1. Introduction

Malaria is a leading cause of morbidity and mortality most especially among vulnerable groups in sub-Saharan Africa. An estimated 228 million cases of malaria occurred worldwide (95% confidence interval (CI): 206–258 million), with 93% of the cases occurring in the World Health Organization (WHO) African Region. Nigeria constitutes one of the six countries that account for more than half of all malaria cases worldwide [1]. While deliberate efforts have resulted in the reduction of malaria deaths, mainly among higher risk groups, the epidemiology and management of malaria in school-age children have received little attention until recently [1, 2]. Studies have shown that reduction in the level of malaria transmission in endemic areas causes gradual immunity acquisition to malaria among older children and adults, thereby making them transmission reservoirs [3, 4]. Recent studies revealed the burden of malaria in school-age children, their role in acting as a reservoir of infection, and the least likelihood of SAC in utilizing insecticide-treated nets (ITNs) [4–6].

The effectiveness of LLITNs in malaria control and the role of schools have been demonstrated [7–11]. While earlier studies have surveyed LLITN usage among school children and school-based net distribution in the integrated national malaria surveillance system [5, 12], there is a paucity of information on the impact of LLITN usage on malaria prevalence among school-age children in Nigeria. This study aims to assess the level of usage of LLITNs and its impact on malaria prevalence among school-age children in Ekiti Southwestern State, Nigeria, with the hope of incorporating the outcome into malaria interventions capable of increasing and sustaining net usage among the study group.

## 2. Materials and Methods

### 2.1. Study Area

The study was carried out among school-age children in Oye and Ikole local government areas (LGAs) in Ekiti State. The State is in one of the three major malaria epidemiological zones in Nigeria and is characterized by a tropical climate with an alternating rainy season (April–October) and dry season (November–March). Temperature ranges between 21°C and 28°C with high humidity, all favourable to malaria vector development.

### 2.2. Sample Size Calculation

Sample size was calculated using the equation *n* = 1.96^2^ pq/L^2^, where *n* = sample size, *p* = expected prevalence (0.5), *q* = 1−p (1–0.5), and *L* = limit.

### 2.3. Study Design

**Figure 1:**
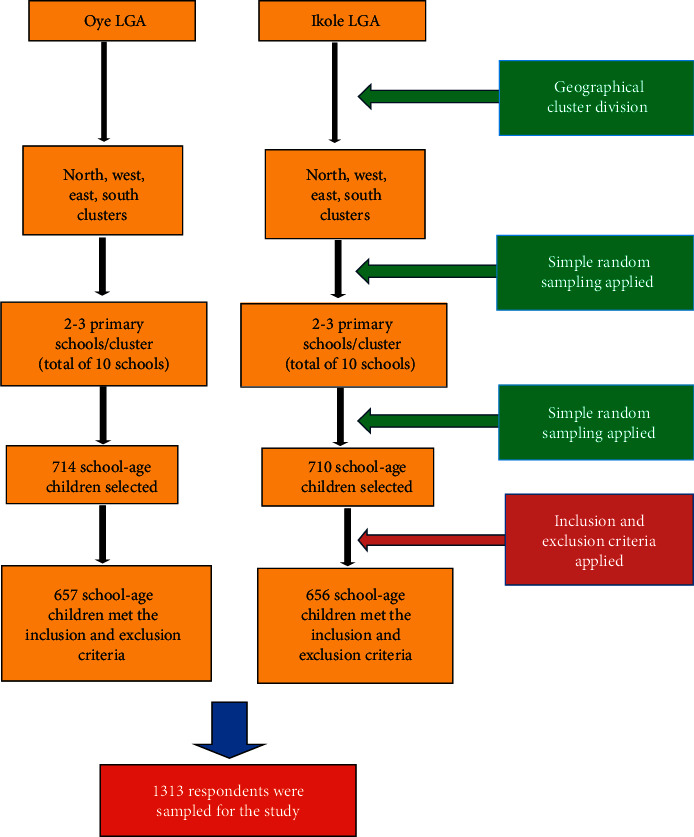
Study flowchart.

A community-based cross-sectional design was used in this study. For this study, only public, mixed primary schools were selected. The study flowchart is shown in Figure 1. Two-stage cluster sampling technique was used given the large size of the study area and based on the availability of resources at the time of the study. Each LGA was divided into geographical clusters of north, east, west, and south. Two to three (2-3) primary schools were then selected from a line list of all primary schools in each of the four (4) clusters using a simple random sampling technique. A total of ten (10) primary schools were selected from the four (4) clusters in each LGA. Simple random sampling technique was then used in selecting 714 and 710 school-age children from Oye and Ikole LGAs, respectively, and out of which 657 and 656 school-age children met the inclusion and exclusion criteria. Finally, a total of 1313 participants from the two (2) LGAs were used for this study (Figure 1).

### 2.4. Data Collection

#### 2.4.1. Data on Malaria Survey

Malaria infection in SAC was diagnosed by using the rapid diagnostic test (RDT) on blood samples that were collected by finger prick from each child. This was conducted and recorded by trained health personnel. The RDTs were the CareStart® Pf/Pv combo test (Access Bio, Inc., Somerset, NJ, USA), containing an alcohol swab, lancet, capillary tube, buffer, and test device.

#### 2.4.2. Questionnaire Administration

A total of 1313 (Oye LGA: 657 and Ikole LGA: 656) semistructured interviewer-guided questionnaires were administered to school-age children who met the inclusion and exclusion criteria in selected primary schools. Sociodemographic information, as well as data on LLITN usage the previous night, was obtained using pretested, semistructured questionnaires adapted from the standardized Malaria Indicator Survey (MIS) tools. Questionnaires were pretested in Ido/Osi local government area of Ekiti State and administered by trained interviewers comprising of fresh university graduates. Data collection occurred during the school term and lasted for two months during May and June 2017. The interviews were conducted in English and Yoruba languages. The interviewers were frequently supervised on the field by a designated team leader and researcher to monitor data collection and provide necessary feedback. Each respondent was interviewed for 20 minutes.

#### 2.4.3. Data Analysis

Data were analyzed using the Statistical Package for Social Sciences (SPSS) for Windows software version 26 (SPSS Inc., Chicago, IL, USA). Frequency tables were generated for relevant variables, and percentages were determined as appropriate. Malaria prevalence was calculated by dividing the number of SAC that have positive RDT result by the total number of pupils that were tested from each LGA. Pearson's chi-square test was used to assess the bivariate association between usage of LLITNs and malaria prevalence. *p* value of less than 0.001 was considered as statistically significant.

### 2.5. Ethical Approval and Consent

Approval for the study was obtained from the Ministry of Health, Ekiti State, MOH/PRS/15/137, Ekiti State Primary Education Board (SPEB), EKSUBEB/ADM/79/123, and Local Government Education Authority Executive Secretaries. Education officers from the district education office in the LGAs assisted the team in locating the schools. The headteacher and teachers at each school that were enrolled in the study were shown approval letters and were informed about the purpose of the study. Awareness and sensitization for the program were carried out through the Parent-Teacher Association (PTA) forum before the study. Only pupils whose parents consented to the study participated in the study.

## 3. Results

### 3.1. Sociodemographic Factors of Respondents

In total, 1313 school children participated in the study: 657 and 656 in selected primary schools in Oye and Ikole LGAs, respectively. The sociodemographic characteristics of the participants are shown in Table 1. Overall mean age was 9.5 ± 2.3. Majority of respondent's mothers completed secondary education (65.3% in Oye LGA; 62.5% in Ikole LGA) and were self-employed (38.7% in Oye LGA; 60.8% in Ikole LGA). Majority were Christians (81.1% in Oye LGA and 85.4% in Ikole LGA), with Yoruba ethnicity (92.8% in Oye LGA and 94.8% in Ikole LGA) with household size ≤4 (54.5% in Oye LGA and 64.3% in Ikole LGA) constituting the majority of respondents in both LGAs (Table 1).

### 3.2. Assessment of the Level of Usage of LLITNs among SAC in Oye and Ikole LGAs

Usage of LLITNs was observed to be generally high in both Oye and Ikole LGAs and was estimated at (79.9%) in Oye LGA and (86.0%) in Ikole LGA, respectively. However, usage of LLITNs was significantly higher in Ikole than Oye LGA (*p* < 0.001) (Table 2).

### 3.3. Association of Sociodemographic Characteristics with Usage of LLITNs among SAC in Oye and Ikole LGAs

There was no significant difference between males and females with respect to the usage of LLITNs in the two LGAs. In Oye LGA ethnicity, household size and access to electricity were observed to have significant association with LLITN usage (*p* < 0.001), while in Ikole LGA, mother's education, mother's occupation, ethnicity, household size, and access to electricity were significantly associated with LLITN usage (*p* < 0.001) (Table 3).

### 3.4. Association of Sociodemographic Characteristics with Malaria Prevalence among SAC in Oye and Ikole LGAs

**Figure 2:**
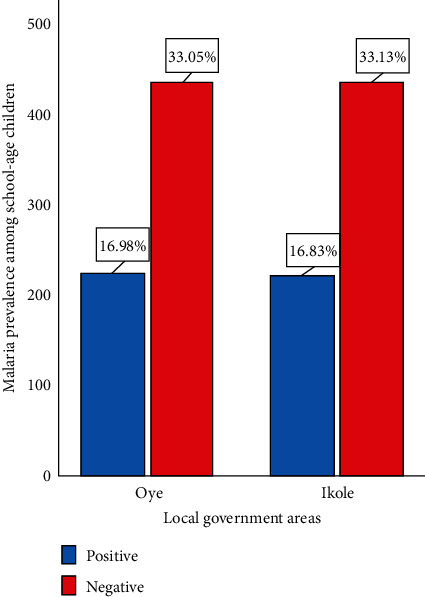
Malaria prevalence among respondents in Oye and Ikole LGAs.

Malaria prevalence in SAC was low in both Oye LGA (16.98%) and Ikole LGA (16.83%) (Figure 2). Sociodemographic factors such as mother's education, mother's occupation, household size, and access to electricity were significantly associated with malaria prevalence in both Oye and Ikole LGAs except in Ikole LGA where additionally, gender showed significance association with malaria prevalence (Table 4).

### 3.5. Relationship between LLITN Usage and Malaria Prevalence among SAC in Oye and Ikole LGAs

The association between LLITN usage and malaria prevalence among SAC in the two LGAs is shown in (Table 5). Our results showed that malaria prevalence was significantly lower among SAC that used LLITNs in both Oye and Ikole LGA (*p* < 0.001) (Table 5).

## 4. Discussion

Vector control through LLITNs constitutes one of the effective control measures in reducing deaths due to malaria [1]. This study aimed at assessing the relationship between the usage of LLITNs and malaria prevalence among school-age children (SAC) in Oye and Ikole LGAs. The findings from this study revealed that the usage of LLITNs was generally high in both LGAs. The rate of LLITN usage observed among SAC in this study is higher in Oye LGA (79.9%) and Ikole LGA (86.0%) than the available record in Kenya, Malawi, and other regions [9, 12–15]. It is, however, lower than the level of usage (88.8%) found in a study in Ibadan [11]. This may be because of the lower age range of most of the respondents (6–8 years) observed in this study as compared with the age range of most of the respondents (10–13 years) found in Ibadan [11]. Studies have shown that higher age increases better reportage [11].

The significant association between household size and LLITN usage observed in this study is consistent with reports from other studies from Ethiopia and Kenya [16–21] where LLITN usage was reportedly high in less household's size than larger household's size. This may be attributed to limited sleeping space in larger households [16, 22]. On the contrary, no significant association was found between LLITN usage and household size in Burkina Faso [23].

Furthermore, the significant association between access to electricity and LLITN usage is consistent with reports from Ethiopia and Tanzania [24, 25]; however, this contradicts another study from South Ethiopia [21].

With regard to mother's education, finding from this study showed that mothers of SAC with secondary education utilized LLITNs in Ikole LGA more than those with primary education. This is consistent with another study from elsewhere [21]. Study has emphasized the role of maternal education in LLITN usage and combating malaria prevalence generally [21]; however, another study found no association [25].

Moreover, LLITN usage was observed to be significantly high among school-age children whose mothers were self-employed. This may be attributed to self-employed mothers having more time for their children's care. This contradicts a report from another study where children of working mothers were observed to have an increased likelihood of LLITN usage [21].

The finding from this study showed that malaria prevalence in the study locations is higher than 10.6% reported in Tanzania [26] and 14% from Gambia [27] but, however, comparable with a prevalence of 17% reported by another author from Gambia [28]. The observed prevalence in this study, however, disagrees with reports from high malaria transmission areas: Uganda [2], Cote d'Ivoire [29], Rwanda [30], and Kenya [31] where higher malaria prevalence has been reported. This may be attributed to the high transmission settings of these areas. The significant association between malaria prevalence and gender is consistent with the report from Cote d'Ivoire [32] and Kenya [31] where high malaria prevalence was observed among males than females but disagrees with a report from elsewhere [26]. The low malaria prevalence observed among school-age children >11 years in this study is consistent with an earlier report from Côte d'Ivoire [32] but disagrees with another report from Kenya [31].

Also, the significant association between malaria prevalence and socioeconomic factors (household size, access to electricity, mother's education, and occupation) agrees with reports from other studies [20, 30, 31].

Furthermore, the low malaria prevalence recorded in Oye and Ikole LGAs in this study may be attributed to the high usage of LLITNs observed among SAC in the study locations. This is consistent with other studies which have reported the effectiveness of net usage in the reduction of malaria cases among school-age children [15, 33–35], but this disagrees with reports from other studies [9, 36].

This study provides baseline information on the utilization of LLITNs among school-age children in Oye and Ikole LGAs and the impact of LLITN usage in reducing malaria infections among the study population.

## 5. Limitations

This is a cross-sectional study, which could only evaluate the association, instead of cause-and-effect relationships. LLITN usage the previous night was used as the basis to determine those who used LLITNs, and this might not be generalizable. The study was conducted in only two LGAs of Ekiti State, and therefore, the results may not be generalized to the country. The study was only based on interviews, and there were no observations conducted to validate the reported use of mosquito nets.

## 6. Conclusion

This study showed a significant association between reduction in malaria infection and usage of LLITNs among school-age children in Oye and Ikole LGAs. This observation requires continuous educations on the usage of LLITNs in the study areas to sustain malaria infection control towards total elimination.

## Figures and Tables

**Table 1:** Sociodemographic characteristics of respondents.

Variables	Oye (%) *N* = 657	Ikole (%) *N* = 656	Total (%) *N* = 1313
*Age (years)*			9.5 ± 2.3
6–8	244 (37.1)	246 (37.5)	490 (37.3)
9–11	246 (37.4)	238 (36.3)	484 (36.9)
≥11	167 (25.5)	172 (26.2)	339 (25.8)

*Gender*			
Male	336 (51.1)	351 (53.5)	687 (52.3)
Female	321 (48.9)	305 (46.5)	626 (47.7)

*Mother's education*			
Primary	102 (15.5)	72 (11.0)	174 (13.3)
Secondary	429 (65.3)	410 (62.5)	839 (63.9)
Tertiary	126 (19.2)	174 (26.5)	300 (22.8)

*Mother's occupation*			
Farmer	69 (10.5)	29 (4.4)	98 (7.5)
Trader	147 (22.4)	31 (4.7)	178 (13.6)
Self-employed	254 (38.7)	399 (60.8)	653 (49.7)
Civil service	168 (25.6)	197 (30.0)	365 (27.8)
Others	19 (2.9)	0 (0.0)	19 (1.4)

*Religion*			
Christian	533 (81.1)	560 (85.4)	1093 (83.2)
Islam	124 (18.9)	96 (14.6)	220 (16.8)

*Ethnicity*			
Yoruba	610 (92.8)	622 (94.8)	1232 (93.8)
Igbo	37 (5.6)	25 (3.8)	62 (4.7)
Hausa	10 (1.5)	9 (1.4)	19 (1.5)

*Household size*			
≤4	358 (54.5)	422 (64.3)	780 (59.4)
>4	299 (45.5)	234 (35.7)	533 (40.6)

*Access to electricity*			
Yes	475 (72.3)	479 (73.0)	954 (72.7)
No	182 (27.7)	177 (27.0)	359 (27.3)

**Table 2:** Assessment of the level of usage of LLITNs among respondents.

Variable	Oye (%) *N* = 657	Ikole (%) *N* = 656	Total (%) *N* = 1313
Usage			
Yes	525 (79.9)	564 (86.0)	1089 (82.9)
No	132 (20.1)	92 (14.0)	224 (17.1)
*p* value	*χ* ^2^ = 8.539, (*p* < 0.001)		

**Table 3:** Association of sociodemographic characteristics with usage of LLITNs in Oye and Ikole LGAs.

Variables	Usage					
	Oye (%)			Ikole (%)		
	Yes *n* = 525	No *n* = 132	Total *N* = 657	Yes *n* = 564	No *n* = 92	Total = 656
*Age*						
6–8	199 (30.3)	45 (6.8)	244 (37.1)	212 (32.3)	34 (5.2)	246 (37.5)
9–11	193 (29.4)	53 (8.1)	246 (37.4)	201 (30.6)	37 (5.60	238 (36.3)
>11	133 (20.2)	34 (5.2)	167 (25.4)	151 (23.0)	21 (3.2)	172 (26.2)
	*χ* ^2^ = 0.744, *p* > 0.001			*χ* ^2^ = 0.936, *p* > 0.001		

*Gender*						
Male	270 (41.1)	66 (10.0)	336 (51.1)	307 (46.8)	44 (6.7)	351 (53.5)
Female	255 (38.8)	66 (10.0)	321 (48.9)	257 (39.2)	48 (7.3)	305 (46.5)
	*χ* = 0.086, *p* > 0.001			*χ* ^2^ = 1.388, *p* > 0.001		

*Mother's education*						
Primary	81 (12.3)	21 (3.2)	102 (15.5)	65 (9.9)	7 (1.1)	72 (11.0)
Secondary	351 (53.4)	78 (11.9)	429 (65.3)	361 (55.0)	49 (7.5)	410 (62.5)
Tertiary	93 (14.2)	33 (5.0)	126 (19.2)	138 (21.1)	36 (5.5)	174 (26.5)
	*χ* ^2^ = 3.909, *p* > 0.001			*χ* ^2^ = 8.978, *p* < 0.001		

*Mother's occupation*						
Farmer	59 (9.0)	10 (1.5)	69 (10.5)	25 (3.8)	4 (0.6)	29 (4.4)
Trader	119 (18.1)	28 (4.3)	147 (22.4)	29 (4.4)	2 (0.3)	31 (4.7)
Self-employed	205 (31.2)	49 (7.5)	254 (38.7)	354 (54)	45 (6.9)	399 (60.8)
Civil service	127 (19.3)	41 (6.2)	168 (25.6)	156 (23.8)	41 (6.3)	197 (30.0)
Others	15 (2.3)	4 (0.6)	19 (2.9)	0 (0.0)	0 (0.0)	
	*χ* ^2^ = 3.506^‡^, *p* > 0.001			*χ* ^2^ = 11.499^‡^, *p* < 0.001		

*Religion*						
Christianity	421 (64.1)	112 (17.0)	533 (81.1)	480 (73.2)	80 (12.2)	560 (85.4)
Islam	104 (15.8)	20 (3.0)	124 (18.9)	84 (12.8)	12 (1.9)	96 (14.6)
	*χ* ^2^ = 1.495, *p* > 0.001			*χ* = 0.217, *p* > 0.001		

*Ethnicity*						
Yoruba	495 (75.3)	115 (17.5)	610 (92.8)	541 (82.5)	81 (12.3)	622 (94.8)
Igbo	29 (4.4)	8 (1.2)	37 (5.6)	19 (2.9)	6 (0.9)	25 (3.8)
Hausa	1 (0.2)	9 (1.4)	10 (1.5)	4 (0.6)	5 (0.8)	9 (1.4)
	*χ* ^2^ = 31.078^‡^, p<0.001			*χ* ^2^ = 15.456^‡^, *p* < 0.001		

*Household size*						
≤4	265 (40.3)	93 (14.2)	358 (54.5)	338 (51.5)	84 (12.8)	422 (64.3)
>4	260 (40.0)	39 (5.9)	299 (45.5)	226 (34.5)	8 (1.2)	234 (35.7)
	*χ* ^2^ = 16.977, *p* < 0.001			*χ* ^2^ = 33.933, *p* < 0.001		

*Access to electricity*						
Yes	360 (54.8)	115 (17.5)	475 (72.3)	393 (60.0)	86 (13.1)	479 (73.0)
No	165 (25.1)	17 (2.6)	182 (27.7)	171 (26.1)	6 (0.9)	177 (27.0)
	*χ* ^2^ = 18.122, *p* < 0.001			*χ* ^2^ = 22.736, *p* < 0.001		

‡ = Fisher's exact test.

**Table 4:** Association of sociodemographic characteristics with malaria prevalence among SAC in Oye and Ikole LGAs.

Variables	Malaria Prevalence					
	Oye (%)			Ikole (%)		
	Yes *n* = 223	No *n* = 434	Total *N* = 657	Yes *n* = 221	No *n* = 435	Total N = 656
*Age*						
6–8	75 (11.4)	169 (25.7)	244 (37.1)	79 (12.0)	167 (25.5)	246 (37.5)
9–11	89 (13.6)	157 (23.9)	246 (37.4)	82 (12.5)	156 (23.8)	238 (36.3)
>11	59 (9.0)	108 (16.4)	167 (25.4)	60 (9.1)	112 (17.1)	172 (26.2)
	*χ* ^2^ = 1.810, *p* > 0.001			*χ* ^2^ = 0.445, *p* > 0.001		

*Gender*						
Male	124 (18.9)	212(32.3)	336 (51.1)	132 (20.1)	219 (33.4)	351 (53.5)
Female	99 (15.1)	222 (33.8)	321 (48.9)	89 (13.6)	216 (32.9)	305 (46.5)
	*χ* ^2^ = 2.692, *p* > 0.001			*χ* ^*2*^ = 5.187, *p* < 0.001		

*Mother's education*						
Primary	30 (4.6)	72 (11.0)	102 (15.5)	31 (4.7)	41 (6.3)	72 (11.0)
Secondary	176 (26.8)	253 (38.5)	429 (65.3)	185 (28.2)	225 (34.3)	410 (62.5)
Tertiary	17 (2.6)	109 (16.6)	126 (19.2)	5 (0.8)	169 (25.8)	174 (26.5)
	*χ* ^2^ = 34.036, *p* < 0.001			*χ* ^2^ = 100.780, *p* < 0.001		

*Mother's occupation*						
Farmer	19 (2.9)	50 (7.6)	69 (10.5)	6 (0.9)	23 (3.5)	29 (4.4)
Trader	46 (7.0)	101 (15.4)	147 (22.4)	10 (1.5)	21 (3.2)	31 (4.7)
Self-employed	113 (17.2)	141 (21.5)	254 (38.7)	205 (31.3)	194 (29.6)	399 (60.8)
Civil service	39 (5.9)	129 (19.6)	168 (25.6)	0 (0)	197 (30.0)	197 (30.0)
Others	6 (0.9)	13 (2.0)	19 (2.9)	0 (0.0)	0 (0.0)	0 (0.0)
	*χ* ^2^ = 22.993, *p* < 0.001			*χ* ^2^ = 158.196, *p* < 0.001		

*Religion*						
Christianity	183 (27.9)	350 (53.3)	533 (81.1)	196 (29.9)	364 (55.5)	560 (85.4)
Islam	40 (6.1)	84 (12.8)	124 (18.9)	25 (3.8)	71 (10.8)	96 (14.6)
	*χ* ^2^ = 0.193, *p* > 0.001			*χ* ^2^ = 2.944, *p* > 0.001		

*Ethnicity*						
Yoruba	209 (31.8)	401 (61.0)	610 (92.8)	211 (32.2)	411 (62.7)	622 (94.8)
Igbo	10 (1.5)	27 (4.1)	37 (5.6)	8 (1.2)	17 (2.6)	25 (3.8)
Hausa	4 (0.6)	6 (0.9)	10 (1.5)	2 (0.3)	7 (1.1)	9 (1.4)
	*χ* ^2^ = 0.981, *p* > 0.001			*χ* ^2^ = 0.577, *p* > 0.001		

*Household size*						
≤4	182 (27.7)	176 (26.8)	358 (54.5)	212 (32.3)	210 (32.0)	422 (64.3)
>4	41 (6.2)	258 (39.3)	299 (45.5)	9 (1.4)	225 (34.3)	234 (35.7)
	*χ* ^2^ = 100.155, *p* < 0.001			*χ* ^2^ = 145.016, *p* < 0.001		

*Access to electricity*						
Yes	222 (33.8)	253 (38.5)	475 (72.3)	221 (33.7)	258 (39.3)	479 (73,0)
No	1 (0.2)	181 (27.6)	182 (27.7)	0 (0)	177 (27.0)	177 (27.0)
	*χ* ^2^ = 125.194, *p* < 0.001			*χ* ^2^ = 123.153, *p* < 0.001		

**Table 5:** Relationship between LLIN usage and malaria test result among respondents.

	Oye LGA		Ikole LGA	
	Positive (%) *N* = 223	Negative (%) *N* = 434	Positive (%) *N* = 221	Negative (%) *N* = 435
LLITN usage				
Yes	171 (26.0)	354 (53.9)	180 (27.4)	384 (58.5)
No	52 (7.9)	80 (12.2)	41 (6.3)	51 (7.8)
*p* value	*χ* ^2^ = 7.638, *p* < 0.001			

## Data Availability

The data used for this study are available from the corresponding author upon request.
